# Identification of major cardiovascular events in patients with diabetes using primary care data

**DOI:** 10.1186/s12913-016-1361-2

**Published:** 2016-04-02

**Authors:** Koen Bernardus Pouwels, Jaco Voorham, Eelko Hak, Petra Denig

**Affiliations:** Unit of PharmacoEpidemiology and PharmacoEconomics, Department of Pharmacy, University of Groningen, Antonius Deusinglaan 1, 9713 AV Groningen, The Netherlands; Department of Clinical Pharmacy and Pharmacology, University Medical Center Groningen, Groningen, The Netherlands

**Keywords:** Cardiovascular diseases, Electronic health records, Diabetes mellitus, Registries, General practice, Sensitivity and specificity

## Abstract

**Background:**

Routine primary care data are increasingly being used for evaluation and research purposes but there are concerns about the completeness and accuracy of diagnoses and events captured in such databases. We evaluated how well patients with major cardiovascular disease (CVD) can be identified using primary care morbidity data and drug prescriptions.

**Methods:**

The study was conducted using data from 17,230 diabetes patients of the GIANTT database and Dutch Hospital Data register. To estimate the accuracy of the different measures, we analyzed the sensitivity, specificity, positive predictive value (PPV), and negative predictive value (NPV) relative to hospitalizations and/or records with a diagnosis indicating major CVD, including ischaemic heart diseases and cerebrovascular events.

**Results:**

Using primary care morbidity data, 43 % of major CVD hospitalizations could be identified. Adding drug prescriptions to the search increased the sensitivity up to 94 %. A proxy of at least one prescription of either a platelet aggregation inhibitor, vitamin k antagonist or nitrate could identify 85 % of patients with a history of major CVD recorded in primary care, with an NPV of 97 %. Using the same proxy, 57 % of incident major CVD recorded in primary or hospital care could be identified, with an NPV of 99 %.

**Conclusions:**

A substantial proportion of major CVD hospitalizations was not recorded in primary care morbidity data. Drug prescriptions can be used in addition to diagnosis codes to identify more patients with major CVD, and also to identify patients without a history of major CVD.

**Electronic supplementary material:**

The online version of this article (doi:10.1186/s12913-016-1361-2) contains supplementary material, which is available to authorized users.

## Background

Routine primary care data are increasingly being used for evaluation and research purposes. In particular, data on drug prescriptions, diagnoses and events are used for (pharmaco)epidemiological and pharmacovigilance studies and for the evaluation of quality of care. Data are becoming more accessible through initiatives, such as European Network of Centres for Pharmacoepidemiology and Pharmacovigilance Resource Database [1]. Validation of information recorded in such databases is required [2]. This is especially relevant in light of upcoming electronic patient record systems, such as the ‘care.data’ scheme in the United Kingdom [3], which are likely to include data from practitioners who are not submitted to rigorous data quality assurance methods.

There is a growing amount of studies evaluating treatment and cardiovascular outcomes using morbidity data from primary care databases [4–8], and also disease cohorts are created using such data [9]. There are concerns, however, about the completeness and accuracy of the diagnoses and events captured in primary care records [10, 11]. Recent research from the UK indicates that a substantial proportion of cardiovascular events is not adequately recorded in primary care morbidity records [12]. Previous studies indicated that adding drug prescriptions may improve the identification of patients with ischemic heart disease (IHD) or myocardial infarction especially when diagnosis recording is poor [13, 14]. Several studies have used drug prescriptions to identify patients with cardiovascular diseases (Additional file 1: Appendix A), but there are questions whether and which drug prescriptions can be used as proxies for identifying patients with prior cardiovascular diagnoses [15]. Previous studies were all from the UK and used Read-codes to identify patients, while the International Classification of Primary Care (ICPC) codes are more widely used across Europe. More information about the validity of morbidity and drug prescription data for identification of cardiovascular events and of prior cardiovascular diseases (CVD) is needed to assess the potential impact of misclassification bias in (pharmaco)epidemiological studies [16, 17]. Given that several studies rely on primary care records alone or solely on drug prescription data, there is a need to evaluate how well CVD events and prior CVD can be identified using these sources of information.

We first evaluated how well major cardiovascular disease (CVD) hospitalizations can be identified from primary care morbidity data and/or drug prescriptions using a Dutch database with type 2 diabetes patients. Secondly, we evaluated the accuracy of different drug proxies to identify patients with a history of major CVD, and to identify a first major CVD event in patients without a history of CVD (incident major CVD).

## Methods

### Study population

This study was conducted using data from the Groningen Initiative to Analyse Type 2 Diabetes Treatment (GIANTT) database [18]. This database contains anonymized data extracted from electronic medical records of type 2 diabetes patients managed by general practitioners in one region in the Netherlands, and includes prescriptions, morbidity, laboratory test results and physical examinations. Morbidity is documented by means of ICPC codes [19] or short text descriptions, which were manually coded in GIANTT. In the Netherlands, each patient is registered with a single GP, who is obliged to keep adequate medical records regarding all relevant diagnostic and prescription information, including out-of-hours prescriptions made by other practitioners. Hence, drug prescriptions were extracted from the same database as the primary care morbidity data.

We included those individuals with data on drug prescriptions available between 1 December 2007 and 1 April 2010, based on enrolment data in GIANTT, who were uniquely linkable with data from the Dutch Hospital Data (DHD) register provided by Statistics Netherlands and survived during the study period (1 January 2008 to 31 December 2009) [20]. The linkage was carried out by Statistics Netherlands, using a match based on gender, date of birth and the 4-digit part of the postal code. Identifying variables were removed by Statistics Netherlands. Overall, 88 % of patients were successfully linked.

### Included hospitalizations, GP diagnoses and medications

Data on hospitalizations were collected from the DHD register provided by Statistics Netherlands [20]. Discharge diagnoses are coded according to the International Classification of Diseases-9-Clinical Modification (ICD-9) and procedures are coded according to the Classification of Medical Procedures developed by the Central Administration of Procedures in the Netherlands. Major CVD included hospitalizations with the following discharge diagnoses or procedures: IHD (ICD-9 code 410-411,413-414), cerebrovascular disease (ICD-9 code 430-437), coronary artery bypass grafting (CABG) or percutaneous transluminal coronary angioplasty (PTCA). Primary care diagnoses and procedures were collected from the GIANTT database. Major CVD included IHD (ICPC code K74-K76), stroke/transient cerebral ischemia (ICPC code K89-K90), coronary artery bypass grafting or percutaneous transluminal coronary angioplasty. Drug prescription data were obtained from the GIANTT database. For the identification of patients with a major CVD, different cardiovascular drug classes were considered based on previous studies (Additional file 1: Appendix A).

### Identification of hospitalization for major CVD

For our first analysis, all major CVD hospitalizations between 1 January 2008 and 31 December 2009 were defined as cases. Information about hospitalizations was obtained from the DHD register. This source has the advantage of providing the most complete and accurately dated information on CVD hospitalizations. We evaluated whether these hospitalizations can be identified using different combinations of primary care morbidity records and cardiovascular drug prescriptions registered during the same period. No restrictions were applied regarding the maximum time between the hospitalization and morbidity record or cardiovascular drug prescription, as long as both were dated within the 2-year period.

### Identification of history of major CVD

We assessed whether patients with a history of major CVD, as documented in the primary care morbidity records (GIANTT data), can be identified using different cardiovascular drug prescriptions. In the Netherlands, primary care records provide the most complete disease history information. Patients with a primary care diagnosis for a major IHD or cerebrovascular event before 1 January 2008 were labeled as patients with a history of major CVD. We evaluated drug prescriptions between 1 January 2008 and 31 December 2008 to estimate the accuracy of different drug proxies. To prevent an underestimation of the specificity, we restricted this analysis to 1 year and excluded patients with an event between 1 January 2008 and 31 December 2008.

### Identification of incident major CVD

We evaluated whether patients with an incident major CVD can be identified using different cardiovascular drug prescriptions. For this, both first major CVD episodes recorded in primary care (GIANTT data) and first major CVD hospitalizations (DHD data) were defined as incident major CVD events. This is expected to provide the most complete information about the occurrence of such events, since some events may not lead to a hospitalization. All first major CVD hospitalizations or primary care diagnosis between 1 January and 31 December 2009 were defined as incident cases. In case of multiple events, the earliest date was used as the index date. This analysis was restricted to 2009, as we were interested in cardiovascular drug treatment initiation, defined as a first prescription of a cardiovascular drug with no prescription of that drug in the previous 365 days. Patients who already had at least one prescription of the drug of interest in the year before 1 December 2008 (allowing a 30 day window prior to incident major CVD events, see below) were excluded. Drug prescriptions prescribed for the first time between 30 days before and 90 days after the index event were considered as true positives, when prescribed more than 30 days before the index event as false positive, and when prescribed more than 90 days after the index event as false negatives. The 30 day before and 90 day after thresholds were used to account for patients receiving their first prescription when scheduled for a coronary revascularization and patients are not being prescribed drugs by their general practitioner during or shortly after hospitalization, respectively.

### Statistical analysis

To estimate the accuracy of the different measures, we analyzed the sensitivity, specificity, positive predictive value (PPV), and negative predictive value (NPV) relative to hospitalizations and/or records with a diagnosis indicating major IHD or cerebrovascular events. Since the priority of which accuracy measure to use largely depends on the research question and goal [21], we report all accuracy measures. Exact binomial 95 % confidence intervals (95 % CI) were calculated. Data were analyzed using SPSS 20 Software and R version 3.0.2.

### Sensitivity analyses

Various sensitivity analyses were performed. First, we repeated analyses for major IHD (ICD-9 410-411, 413-414, CABG and PTCA vs ICPC K74-K76, CABG and PTCA) and cerebrovascular disease (ICD-9 430-437 vs ICPC K89-K90) separately. Second, we evaluated whether the accuracy measures would improve when comparing myocardial infarction hospitalizations (ICD-9 code 410), as a well-defined endpoint, with the primary care codes for myocardial infarction or chronic ischemic heart disease, which includes past myocardial infarction (ICPC codes K75-K76). Similarly, we evaluated accuracy measures for well-defined cerebrovascular events/stroke codes (ICD-9 430-434 vs ICPC K90). Third, we tested the impact of possible registration problems at GP and DHD level. For this, we excluded 18 GPs with a relatively low morbidity registration, and 63 additional GPs with a possible delay in registration or situated in an area for which the closest hospital did not provide complete data to the DHD register during the entire study period. Fourth, we explored the influence of using at least two or three prescriptions within 1 year to identify patients with a history of major CVD instead of at least one prescription within a year. Fifth, we compared the concordance between major CVD hospitalizations and primary care morbidity records in patients with and without a history of major CVD. This sensitivity analysis was performed to assess whether GPs are less likely to re-enter the diagnostic code in case of a hospitalization for patients with a history of major CVD.

### Ethics statement

In The Netherlands, according to the Code of Conduct for the use of data in Health Research (“Gedragscode gezondheidsonderzoek” approved in 2004 by the Dutch College for Protection of Personal Data, taking into account Article 25 of the Dutch Act on the Protection of Personal Data) no ethics committee approval was required for this research using data from anonymous medical records.

## Results

### Study population

A cohort of 17,230 patients with type 2 diabetes was eligible for analyses. At baseline, mean age of the study population was 66 years (sd 12), 48 % were men, median diabetes duration was 6 years (interquartile range: 3–10) and the prevalence of at least one major CVD diagnosis recorded by a GP was 16 %.

### Identification of hospitalization for major CVD

Between January 2008 and December 2009, 729 (4 %) patients were hospitalized for a major CVD event or procedure. The primary care diagnoses recorded in the same period had a sensitivity of 43 % and a PPV of 35 % for identifying major CVD hospitalizations (Table 1). Adding nitrate prescriptions to the search resulted in a sensitivity of 68 % and a PPV of 24 %. A proxy based on primary care diagnoses, platelet aggregation inhibitor, vitamin k antagonist or nitrate prescriptions had a sensitivity of 94 % and PPV of 10 %. Major cerebrovascular hospitalizations were more often identified using primary care diagnoses alone than major CVD hospitalizations (57 % vs 38 %). When only considering myocardial infarction hospitalizations, the sensitivity of primary care diagnoses was with 46 % still lower than for cerebrovascular hospitalizations (57 %). Restricting the analysis to only well-defined cerebrovascular events decreased the sensitivity from 57 to 54 %.

**Table 1:** Identifying major IHD or cerebrovascular hospitalizations

GP codes^a^/drug (at least 1 prescription)	TP	FP	TN	FN	Sens (95 % CI)	Spec (95 % CI)	PPV (95 % CI)	NPV (95 % CI)
IHD/cerebrovascular hospitalizations								
GP codes	312	567	15934	417	43 (39–46)	97 (96–97)	35 (32–39)	97 (97–98)
GP codes/nitrates	498	1602	14899	231	68 (65–72)	90 (90–91)	24 (22–26)	98 (98–99)
GP codes/nitrates/platelet aggregation inhibitors	672	5123	11378	57	92 (90–94)	69 (68–70)	12 (11–12)	100 (99–100)
GP codes/nitrates/platelet aggregation inhibitors/vitamin k antagonists	685	6297	10204	44	94 (92–96)	62 (61–63)	10 (9–11)	100 (99–100)
IHD hospitalizations								
GP codes	212	431	16240	347	38 (34–42)	97 (97–98)	33 (29–37)	98 (98–98)
GP codes/nitrates	391	1499	15172	168	70 (66–74)	91 (91–91)	21 (19–23)	99 (99–99)
GP codes/nitrates/platelet aggregation inhibitors	520	5217	11454	39	93 (91–95)	69 (68–69)	9 (8–10)	100 (100–100)
GP codes/nitrates/platelet aggregation inhibitors/vitamin k antagonists	529	6421	10250	30	95 (92–96)	61 (61–62)	8 (7–8)	100 (100–100)
Cerebrovascular hospitalizations								
GP codes	105	172	16874	79	57 (50–64)	99 (99–99)	38 (32–44)	100 (99–100)
GP codes/platelet aggregation inhibitors	161	5162	11884	23	88 (82–92)	70 (69–70)	3 (3–4)	100 (100–100)
GP codes/platelet aggregation inhibitors/vitamin k antagonists	168	6603	10443	16	91 (86–95)	61 (61–62)	2 (2–3)	100 (100–100)

*Abbreviations: IHD* ischaemic heart disease, *GP* general practitioner, *TP* true positive, *FP* false positive, *TN* true negative, *FN* false negative, *Sens* sensitivity, *Spec* specificity, *PPV* positive predictive value, *NPV* negative predictive value

^a^GP codes are the following primary care diagnoses: angina pectoris (ICPC code K74), myocardial infarction (ICPC code K75), other or chronic ischemic heart disease (ICPC code K76), transient cerebral ischemia (ICPC code K89), stroke or cerebrovascular accident (ICPC code K90), coronary artery bypass grafting or percutaneous transluminal coronary angioplasty

Results were similar for patients with and without a history of major CVD recorded in primary care morbidity data prior January 2008, e.g. for major CVD hospitalizations the sensitivity was 42 % for patients without and 45 % for patients with a history of major CVD.

### Identifying history of major CVD

Next, we assessed whether patients with a history of major CVD (15 % of the included patients) - as documented in primary care morbidity records - can be identified using different cardiovascular drug prescriptions. The sensitivity of 1 prescription of individual drugs ranged between 1 % for nicotinic acid and derivatives and 70 % for platelet aggregation inhibitors (Table 2 and Additional file 1: Appendix C). When at least one prescription of either a platelet aggregation inhibitor, a vitamin k antagonist or nitrate was used as a proxy, the sensitivity increased to 85 %. When considering only a history of major CVD, this proxy had a 100 % sensitivity (Table 2). The specificity of one prescription of individual drugs ranged between 36 % for statins and 100 % for nicotinic acid and derivatives (Additional file 1: Appendix C). The proxy including three had a specificity of 75 %. PPVs for individual drugs ranged between 15 % for thiazides and 52 % for nitrates (Additional file 1: Appendix C). The afore mentioned proxy including three drug classes had a PPV of 37 %. NPVs were equal to or above 85 % for all drug proxies.

**Table 2:** Identifying a history of major IHD or cerebrovascular disease using GP diagnoses as a reference standard

Drug (at least 1 prescription)	TP	FP	TN	FN	Sens (95 % CI)	Spec (95 % CI)	PPV (95 % CI)	NPV (95 % CI)
IHD/cerebrovascular history								
Vitamin K antagonists	424	1029	12996	2010	17 (16–19)	93 (92–93)	29 (27–32)	87 (86–87)
Platelet aggregation inhibitors	1696	2500	11525	738	70 (68–72)	82 (82–83)	40 (39–42)	94 (94–94)
Nitrates	487	441	13584	1947	20 (18–22)	97 (97–97)	52 (49–56)	87 (87–88)
Vitamin K antagonists/Platelet aggregation inhibitors	2045	3441	10584	389	84 (83–85)	75 (75–76)	37 (36–39)	96 (96–97)
Vitamin K antagonists/Platelet aggregation inhibitors/Nitrates	2077	3519	10506	357	85 (84–87)	75 (75–76)	37 (36–38)	97 (96–97)
IHD history								
Vitamin K antagonists	354	1099	13453	1553	19 (17–20)	92 (92–93)	24 (22–27)	90 (89–90)
Platelet aggregation inhibitors	1312	2884	11668	595	69 (67–71)	80 (80–81)	31 (30–33)	95 (95–96)
Nitrates	466	462	14090	1441	24 (23–26)	97 (97–97)	50 (47–53)	91 (90–91)
Vitamin K antagonists/Platelet aggregation inhibitors	1603	3883	10669	304	84 (82–86)	73 (73–74)	29 (28–30)	97 (97–98)
Vitamin K antagonists/Platelet aggregation inhibitors/Nitrates	1907	3961	10591	0	100 (100–100)	73 (72–74)	32 (31–34)	100 (100–100)
Cerebrovascular history								
Vitamin K antagonists	119	1334	14424	582	17 (14–20)	92 (91–92)	8 (7–10)	96 (96–96)
Platelet aggregation inhibitors	506	3690	12068	195	72 (69–75)	77 (76–77)	12 (11–13)	98 (98–99)
Nitrates	67	861	14897	634	10 (7–12)	95 (94–95)	7 (6–9)	96 (96–96)
Vitamin K antagonists/Platelet aggregation inhibitors	604	4882	10876	97	86 (83–89)	69 (68–70)	11 (10–12)	99 (99–99)
Vitamin K antagonists/Platelet aggregation inhibitors/Nitrates	604	4992	10766	97	86 (83–89)	68 (68–69)	11 (10–12)	99 (99–99)

*Abbreviations: IHD* ischaemic heart disease, *GP* general practitioner, *TP* true positive, *FP* false positive, *TN* true negative, *FN* false negative, *Sens* sensitivity, *Spec* specificity, *PPV* positive predictive value, *NPV* negative predictive value

Results were similar when using two or more and three or more prescriptions within 1 year as a requirement for a positive test (Additional file 1: Appendix D).

### Identifying incident major CVD

Using primary care data or hospitalizations indicating incident major CVD as a reference, only 13 % of incident major CVD events could be identified using nitrate prescriptions alone (Table 3). However, a proxy based on one prescription of either a platelet aggregation inhibitor, a vitamin k antagonist or nitrate identified 57 % of incident major CVD events. This proxy had a specificity of 94 % and a PPV of 17 %.

**Table 3:** Identifying incident major IHD or cerebrovascular events^a^

Drug (at least 1 prescription)	TP	FP	TN	FN	Sens (95 % CI)	Spec (95 % CI)	PPV (95 % CI)	NPV (95 % CI)
GP/Hosp IHD/cerebrovascular events^b^								
Vitamin K antagonists	16	324	14689	569	3 (2–4)	98 (98–98)	5 (3–8)	96 (96–97)
Platelet aggregation inhibitors	147	495	11654	167	47 (41–53)	96 (96–96)	23 (20–26)	99 (98–99)
Nitrates	73	308	15196	470	13 (11–17)	98 (98–98)	19 (15–23)	97 (97–97)
Vitamin K antagonists/Platelet aggregation inhibitors	129	626	10230	106	55 (48–61)	94 (94–95)	17 (14–20)	99 (99–99)
Vitamin K antagonists/Platelet aggregation inhibitors/Nitrates	132	650	10088	98	57 (51–64)	94 (93–94)	17 (14–20)	99 (99–99)
Hosp IHD/cerebrovascular events^c^								
Vitamin K antagonists	13	331	14944	310	4 (2–7)	98 (98–98)	4 (2–6)	98 (98–98)
Platelet aggregation inhibitors	107	545	11744	68	61 (53–68)	96 (95–96)	16 (14–19)	99 (99–100)
Nitrates	59	330	15431	227	21 (16–26)	98 (98–98)	15 (12–19)	99 (98–99)
Vitamin K antagonists/Platelet aggregation inhibitors	92	673	10282	44	68 (59–75)	94 (93–94)	12 (10–15)	100 (99–100)
Vitamin K antagonists/Platelet aggregation inhibitors/Nitrates	94	697	10137	39	71 (62–78)	94 (93–94)	12 (10–14)	100 (99–100)

*Abbreviations: IHD* ischaemic heart disease, *GP* general practitioner, *Hosp* hospitalization, *TP* true positive, *FP* false positive, *TN* true negative, *FN* false negative, *Sens* sensitivity, *Spec* specificity, *PPV* positive predictive value, *NPV* negative predictive value

^a^Prescriptions are considered to be in agreement with actual events if being prescribed between 30 days before to 90 days after the date of the major IHD or cerebrovascular event

^b^Using hospitalizations or general practitioners diagnoses as reference standard

^c^Using only hospitalizations as reference standard

When using only hospitalizations as a reference, the sensitivity of this proxy increased to 71 %, while the specificity and PPV were 94 and 12 %, respectively.

Removing patients from GPs with possible registration problems did not alter the findings substantially (Additional file 1: Appendices B, E and F).

## Discussion

Only 43 % of major CVD hospitalizations could be identified using diagnoses recorded in primary care in the same period. Adding drug prescriptions to the search increased the sensitivity up to 94 %. The proxy of at least one prescription of either a platelet aggregation inhibitor, a vitamin k antagonist or nitrate prescription could identify 85 % of patients with a history of major CVD recorded in primary care. With this drug prescription proxy, also 57 % of the incident major CVD recorded in either primary or hospital care records could be identified.

Our finding that less than half of the hospitalizations for a major CVD were identified using primary care morbidity records indicates that even for major events such records are incomplete. Focusing only on myocardial infarction slightly increased this figure to 46 %. For comparison, it was estimated that approximately 25 % of myocardial infarctions were not recorded in primary care morbidity records of the widely researched Clinical Practice Research Datalink (CPRD) [12]. For some of the included morbidity codes, it is possible that general practitioners do not immediately enter a new diagnosis in their system after receiving a discharge letter from the hospital. The CPRD study found that in primary care records the discharge date is often used to record myocardial infarctions [12], which may especially for hospitalizations with a long duration result in misclassification of the date of the event. In addition, some patients may already have a history of major CVD which theoretically may reduce the likelihood to re-enter the diagnostic code in case of a hospitalization. However, our results were similar for patients with and without a history of major CVD recorded in primary care morbidity data. Therefore, adding drug proxies to morbidity codes may be useful to identify patients with major CVD when the quality of diagnosis coding is poor [14].

The selected drugs had a relatively high specificity to identify both a history of major CVD and incident CVD, although some of the evaluated drugs may be prescribed for other indications. For example, a Dutch study found that 41 % of 8,718 patients receiving antiplatelet therapy had only a non-recommended cardiovascular indication (*n* = 982) or related cardiometabolic disease (*n* = 2,557) recorded, suggesting frequent use of these drugs for primary prevention [22]. In addition, some of the evaluated drugs are used for other indications, such as venous thrombosis. Previous research suggested that nitrate prescriptions can be used to identify patients with CVD [13, 14, 23]. Our study confirms that nitrates indeed have the highest specificity to identify such patients but the sensitivity is lower than found in the other studies (24 % vs 47–55 %) [13, 14]. This difference may be due to the fact that we used a broader reference standard, that is, all patients with acute angina pectoris, myocardial infarction, chronic ischemic heart disease, CABG or PTCA recorded in primary care morbidity data. Donnan et al. evaluated whether patients with a myocardial infarction could be identified using nitrate prescriptions [14], while Gray et al. evaluated whether nitrates could be used to identify patients with ischemic heart disease, including probable cases defined as patients with a written record strongly suggesting ischemic heart disease and receiving drugs that could be used to treat angina [13]. As nitrates, which are frequently used to treat angina, were consequently used to create the golden standard in that study, one would expect a higher sensitivity than in our study.

Both for identifying a history of CVD and incident major CVD events, a combined drug proxy including also vitamin K antagonists and platelet aggregation inhibitors had a much higher sensitivity than nitrates alone, with some loss in specificity. As already pointed out earlier by McManus et al., strokes may be difficult to identify using drug prescriptions [24]. Approximately 10 % of strokes are due to hemorrhagic strokes [25, 26], which are particularly difficult to identify using drug prescriptions. This may explain why a history of major CVD events could be better identified using drug prescriptions than a history of major cerebrovascular events, despite the fact that major cerebrovascular hospitalizations were better registered in primary care morbidity records.

In general, the PPVs for identification of patients with major CVD were low. This is partly because the majority of patients did not have a major CVD diagnosis and may have other indications for which the selected drugs can be prescribed, such as venous thrombosis [22]. To identify as many patients with major CVD as possible from primary care records one needs a search strategy with a high sensitivity, thus using both diagnosis codes and drug proxies. On the other hand, for selecting a cohort of primary prevention patients, a high NPV is important. Our study illustrates that using drug proxies only can be adequate with a NPV of 97 %, in a population with a similar prevalence of major CVD.

This study has some important strengths. This is the first study that evaluates the accuracy of a wide range of primary care diagnoses and drug prescriptions for identification of patients with major CVD. We evaluated the accuracy of general practice morbidity records using ICPC codes supplemented with diagnoses obtained from verbal descriptions, thereby capturing more major CVD events in general practice morbidity records then when solely relying on diagnostic codes. Moreover, ICPC codes are more widely used across Europe than the Read codes that were used in most previous validation studies. In addition, we evaluated whether incident and a history of any major CVD could be identified, while most previous studies evaluated only specific cardiovascular outcomes, such as myocardial infarction [14], angina [27] or atherothrombosis [22].

This study has also some limitations. Underlying most studies assessing the validity of search algorithms to identify patients with a specific disease is the lack of a true golden standard registry. This limitation is particularly relevant when evaluating the validity of drug proxies for the identification of patients with CVD in primary care records. As we found that only 43 % of major CVD hospitalizations were recorded in primary care morbidity data, it can be expected that the specificity of the different drug proxies are underestimates of the true values. Furthermore, some events identified using primary care morbidity data may be minor events not requiring hospitalization or working hypotheses. Despite the lack of a golden standard, we preferred presenting accuracy measures like sensitivity, specificity, positive predictive value and negative predictive value over a single agreement measure like kappa, because these accuracy measures provide better insight about, for example, what percentage of hospitalizations is not in primary care morbidity records. We presented data for patients managed in primary care in the Northern Netherlands, which may limit the generalizability of our findings. Individual practices, practices from other regions or countries may register morbidity worse or better and may differ in their prescribing habits. Also, prescribing habits may differ for specific subpopulations. On the other hand, our analyses based on GIANTT data represent 80 % of all general practices in the region. Therefore, the data provide a ‘real-world’ picture of GPs using electronic health-care records in the community. In contrast, practices included in special registration networks with rigorous data quality assurance methods may have better morbidity registration.

We only included patient surviving the whole study period and hence could not assess fatal events or concordance in patients that died within the study period. Hence, results should not be generalized for studying fatal CVD events. On the other hand, fatal major CVD events are less relevant when selecting a cohort of patients with or without a history of major CVD or identifying more patients than with diagnosis codes alone, i.e. the situations where drug proxies are often used.

Finally, we had to exclude 12 % of patients who were not uniquely identifiable in the Dutch Hospital Data register for the whole study period. Not all hospitals provide complete data to the DHD register during the entire study period. However, removing patients from practices in the area for which the closest hospital did not provide complete data to DHD did not substantially influence the results.

In future projects, the accuracy and completeness of diagnoses in electronic medical records may be improved by investigating free text parts using text-mining techniques [28–30]. Furthermore, there are also initiatives to improve recording of diagnoses in electronic medical records [31, 32]. The finding that a large proportion of major CVD hospitalizations is not recorded in primary care morbidity data is particularly relevant in the context of upcoming regional and nationwide electronic healthcare databases.

## Conclusions

In conclusion, a substantial proportion of major CVD hospitalizations was not recorded in primary care morbidity data. This stresses the importance of combining different types of data and linkage of different data sources to identify patients with incident or prevalent diseases. Using drug prescriptions as proxies may help to increase the identification of patients with major CVD. Moreover, although it appeared difficult to identify patients with incident major CVD using only drug prescriptions, it seems feasible to select a cohort of patients without a history of major CVD using a proxy of at least 1 prescription of either a platelet aggregation inhibitor, a vitamin k antagonist or nitrate.

## Additional file

Additional file 1: Appendix A. Table A1. Studies using or assessing the validity of drug proxies to identify different cardiovascular diseases. Appendix B. Table B1. Identifying major CVD hospitalizations^a^. Appendix C. Table C1. Identifying a history of major CVD using GP diagnoses as a reference standard. Appendix D. Table D1. Identifying a history of major CVD using GP diagnoses as a reference standard^a^. Appendix E. Table E1. Identifying a history of major CVD using GP diagnoses as a reference standard^a^. Appendix F. Table F1. Identifying incident major CVD within -30 to 90 days of event^a,b^. (DOCX 45 kb)

## Abbreviations

CABG: coronary artery bypass grafting
CI: confidence intervals
CPRD: clinical practice research datalink
CVD: cardiovascular disease
DHD: Dutch hospital data
GIANTT: Groningen initiative to analyse type 2 diabetes treatment
GP: general practitioner
ICD-9: international classification of diseases-9-clinical modification
ICPC: international classification of primary care
IHD: ischemic heart disease
NPV: negative predictive value
PPV: positive predictive value
PTCA: percutaneous transluminal coronary angioplasty
sd: standard deviation

## References

[1] European Network of Centres for Pharmacoepidemiology and Pharmacovigilance. ENCePP Home Page. www.encepp.eu. Accessed 18 Aug 2015.

[2] Hall GC, Sauber B, Bourke A, Brown JS, Reynolds MW, LoCasale R (2012). Guidelines for good database selection and use in pharmacoepidemiology research. Pharmacoepidemiol Drug Saf.

[3] NHS England. The care.data programme – better information means better care. http://www.england.nhs.uk/ourwork/tsd/care-data/. Accessed 3 Nov 2014.

[4] Sidorenkov G, Voorham J, de Zeeuw D, Haaijer-Ruskamp FM, Denig P (2013). Do treatment quality indicators predict cardiovascular outcomes in patients with diabetes?. PLoS One.

[5] Loke YK, Kwok CS, Singh S (2011). Comparative cardiovascular effects of thiazolidinediones: systematic review and meta-analysis of observational studies. BMJ.

[6] Johnson JA, Majumdar SR, Simpson SH, Toth EL. Decreased mortality associated with the use of metformin compared with sulfonylurea monotherapy in type 2 diabetes. Diabetes Care. 2002;25:2244–8. 10.2337/diacare.25.12.2244 12453968

[7] Pouwels KB, van Grootheest K (2012). The rosiglitazone decision process at FDA and EMA. What should we learn?. Int J Risk Saf Med.

[8] Roumie CL, Greevy RA, Grijalva CG, Hung AM, Liu X, Murff HJ (2014). Association between intensification of metformin treatment with insulin vs sulfonylureas and cardiovascular events and all-cause mortality among patients with diabetes. JAMA.

[9] Wells S, Riddell T, Kerr A, Pylypchuk R, Chelimo C, Marshall R, Exeter DJ, Mehta S, Harrison J, Kyle C, Grey C, Metcalf P, Warren J, Kenealy T, Drury PL, Harwood M, Bramley D, Gala G, Jackson R. Cohort Profile: The PREDICT Cardiovascular Disease Cohort in New Zealand Primary Care (PREDICT-CVD 19). Int J Epidemiol. 2015. [Epub ahead of print] 10.1093/ije/dyv312 26686841

[10] Moher M, Yudkin P, Turner R, Schofield T, Mant D (2000). An assessment of morbidity registers for coronary heart disease in primary care. ASSIST (ASSessment of implementation STrategy) trial collaborative group. Br J Gen Pract.

[11] Payne RA, Abel GA, Simpson CR (2012). A retrospective cohort study assessing patient characteristics and the incidence of cardiovascular disease using linked routine primary and secondary care data. BMJ Open.

[12] Herrett E, Shah AD, Boggon R, Denaxas S, Smeeth L, van Staa T, Timmis A, Hemingway H (2013). Completeness and diagnostic validity of recording acute myocardial infarction events in primary care, hospital care, disease registry, and national mortality records: Cohort study. BMJ.

[13] Gray J, Majeed A, Kerry S, Rowlands G (2000). Identifying patients with ischaemic heart disease in general practice: Cross sectional study of paper and computerised medical records. BMJ.

[14] Donnan PT, Dougall HT, Sullivan FM (2003). Optimal strategies for identifying patients with myocardial infarction in general practice. Fam Pract.

[15] Cossman RE, Cossman JS, James WL, Blanchard T, Thomas RK, Pol LG, Cosby AG, Mirvis DM (2008). Evaluating heart disease presciptions-filled as a proxy for heart disease prevalence rates. J Health Hum Serv Adm.

[16] Copeland KT, Checkoway H, McMichael AJ, Holbrook RH (1977). Bias due to misclassification in the estimation of relative risk. Am J Epidemiol.

[17] Pekkanen J, Sunyer J, Chinn S (2006). Nondifferential disease misclassification may bias incidence risk ratios away from the null. J Clin Epidemiol.

[18] Voorham J, Denig P (2007). Computerized extraction of information on the quality of diabetes care from free text in electronic patient records of general practitioners. J Am Med Inform Assoc.

[19] Lamberts W, Woods M (1987). ICPC: International Classification of Primary Care.

[20] Statistics Netherlands. Microdata services: Conduct your own research using data from Statistics Netherlands. http://www.cbs.nl/en-GB/menu/informatie/beleid/zelf-onderzoeken/default.htm. Accessed 10 Sept 2014.

[21] Chubak J, Pocobelli G, Weiss NS (2012). Tradeoffs between accuracy measures for electronic health care data algorithms. J Clin Epidemiol.

[22] Van de Steeg-van Gompel CH, Wensing M, Braspenning J, De Smet PA (2012). The usefulness of antiplatelet prescriptions for the identification of patients with atherothrombosis in primary care: A Dutch cross-sectional study. J Eval Clin Pract.

[23] Thiru K, Donnan PT, Weller P, Sullivan F (2009). Identifying the optimal search strategy for coronary heart disease patients in primary care electronic patient record systems. Inform Prim Care.

[24] McManus RJ, Lumley L, Gough M, Jhass L, Deacon K (2001). New beginning for care for elderly people? framework will have considerable effect on primary care. BMJ.

[25] Andersen KK, Olsen TS, Dehlendorff C, Kammersgaard LP (2009). Hemorrhagic and ischemic strokes compared: Stroke severity, mortality, and risk factors. Stroke.

[26] Berger K, Ajani UA, Kase CS, Gaziano JM, Buring JE, Glynn RJ, Hennekens CH (1999). Light-to-moderate alcohol consumption and risk of stroke among U.S. male physicians. N Engl J Med.

[27] der Zee AH M-v, Klungel OH, Stricker BH, van der Kuip DA, Witteman JC, Hofman A (2003). Repeated nitrate prescriptions as a potential marker for angina pectoris. A comparison with medical information from the Rotterdam Study. Pharm World Sci.

[28] Pakhomov S, Bjornsen S, Hanson P, Smith S (2008). Quality performance measurement using the text of electronic medical records. Med Decis Making.

[29] Warrer P, Hansen EH, Juhl-Jensen L, Aagaard L (2012). Using text-mining techniques in electronic patient records to identify ADRs from medicine use. Br J Clin Pharmacol.

[30] Schuemie MJ, Sen E, ‘t Jong GW, van Soest EM, Sturkenboom MC, Kors JA (2012). Automating classification of free-text electronic health records for epidemiological studies. Pharmacoepidemiol Drug Saf.

[31] The Dutch College of General Practitioners (Nederlands Huisartsen Genootschap (NHG)). Digital Program Individual In-Service Training ‘Adequate record keeping in electronic patient records’ (Digitaal Programma Individuele Nascholing (PIN) over Adequate Dossiervorming met het Electronisch Patientendossier (ADEPD)) https://www.nhg.org/actueel/nieuws/gratis-pin-e-learning-over-adepd. Accessed 18 Aug 2015.

[32] Jabaaij L, Njoo K, Visscher S, Van den Hoogen H, Tiersma W, Levelink H (2009). Improve your records – use the EPD-scan-h. Huisarts Wet.

