# Supplementary material for: Identification of major cardiovascular events in patients with diabetes using primary care data
Source: BMC Health Serv Res. 2016 Apr 2;16:110. doi: 10.1186/s12913-016-1361-2 (PMC4818875; doi:10.1186/s12913-016-1361-2)
Supplement: Additional file 1: — Appendix A. Table A1. Studies using or assessing the validity of drug proxies to identify different cardiovascular diseases. Appendix B. Table B1. Identifying major CVD hospitalizationsa. Appendix C. Table C1. Identifying a history of major CVD using GP diagnoses as a reference standard. Appendix D. Table D1. Identifying a history of major CVD using GP diagnoses as a reference standarda. Appendix E. Table E1. Identifying a history of major CVD using GP diagnoses as a reference standarda. Appendix F. Table F1. Identifying incident major CVD within -30 to 90 days of eventa,b. (DOCX 45 kb) [file 12913_2016_1361_MOESM1_ESM.docx]

**Appendix A.**

**Table A1. Studies using or assessing the validity of drug proxies to identify different cardiovascular diseases**

| **Author** | **Country** | **Disease** | **Drug proxy (ATC code)** |
| --- | --- | --- | --- |
| Larsen et al [1] | Denmark | Cardiovascular disease history | At least one prescription of: antithrombotics (B01), cardiac glycosides (C01), antiarrhytmics, nitrates (C01), antihypertensives (C02), diuretics (C03), beta-blockers (C07), calcium antagonists (C08) or ACE inhibitors (C09) |
| Abraha et al [2] | Italy | Cardiovascular disease history | At least one prescription of:  Antithrombotics (B01), cardiac glycosides, antiarrhytmics, nitrates (C01), antihypertensives (C02), diuretics (C03), beta-blockers (C07), calcium antagonists (C08), ACE inhibitors (C09) or low-dose aspirin |
| Biagi et al [3] | Italy | Cardiovascular disease history | At least one prescription of:  Cardiac therapy (C01), antihypertensives (C02), diuretics (C03), beta-blocking agents (C07), calcium-channel blockers (C08), agents acting on the renin-angiotensin system (C09) or lipid-modifying agents (C10) |
| Christiansen et al [4] | Denmark | Previous atrial fibrillation or flutter | Prescriptions of digoxin or vitamin K antagonists |
| Christiansen et al [4] | Denmark | Cardiovascular disease history | Prescriptions of angiotensin-converting enzyme inhibitors, beta-blocking agents, low-dose aspirin, statins, calcium antagonists, other antihypertensives, diuretics or nitrates |
| Friis et al [5] | Denmark | Cardiovascular disease | Prescriptions of oral anticoagulants (B01AA03, B01AA04), cardiac glycosides (C01A), anti-arrhytmics (C01B), cardiac stimulants (C01C), vasodilators (C01D), beta-blocking agents (C07), calcium channel blockers (C08), angiotensin-converting enzyme inhibitors (C09A, C09B) or angiotensin II antagonists (C09C, C09D) |
| Latry et al [6] | France | Cardiovascular disease | Prescriptions of central antihypertensives, beta-blockers, diuretics, calcium channel blockers, ACE inhibitors or angiotensin II receptor antagonists |
| Bak et al [7,8] | Denmark | Hypertension, cardial arrhythmia and ischemic heart disease | Prescription of diuretics (C03), beta-blockers (C07), calcium channel blockers (C08), angiotensin-converting enzyme inhibitors (C09), antiarrhytmics (C01A, C01B), or antianginal drugs (C01D) |
| Maitland-van der Zee et al [9] | Netherlands | Angina pectoris | At least two prescriptions of nitrates |
| Filippi et al [10] | Italy | Angina | At least two prescriptions of nitrates |
| Williams et al [11] | Ireland | Ischaemic heart disease | Prescription of nitrate (C01DA) |
| Johnson et al [12] | Canada | Established coronary disease | Use of nitrates |
| Teeling et al [13] | Ireland | Presumed ischaemic heart disease | Coprescription of aspirin (B01AC06, N02BA01) and nitrate (C01DA) |
| Wan Md Adnan et al [14] | Ireland | Ischaemic heart disease | Three or more prescriptions of nitrate (C01DA) or nicorandil (C01DX16) with aspirin (B01AC06, N02BA01) |
| Wan Md Adnan et al [14] | Ireland | Heart failure | Three or more prescriptions of loop diuretics (C03C) |
| Wan Md Adnan et al [14] | Ireland | Hypertension | Three or more prescriptions of beta-adrenoceptor blockers (C07), diuretics (C03A, C03B) or calcium channel blockers (C07) |
| Rasmussen et al [15] | Denmark | Heart failure | At least one prescription of loop diuretics (C03C) |
| Meijvis et al [16] | Netherlands | Congestive heart failure | Two or more prescriptions of digoxin with diuretics |
| Van de Steeg-van Gompel et al [17] | Netherlands | Atherothrombosis | At least one prescription of platelet aggregation inhibitors (B01AC) |
| Cossman et al [18] | United States | Heart disease | Prescriptions of renin angiotensin systemic antagonists, beta and alpha blockers or cholesterol reducers and lipotropics |
| Gray et al [19] | United Kingdom | Ischemic heart disease | Various combinations of prescriptions of nitrate, aspirin, atenolol, statin and digoxin |
| Donnan et al [20] | United Kingdom | Myocardial infarction | Prescription of nitrates, aspirin, statins or beta-blockers |
| De Vries et al [21] | Netherlands | History of cardiovascular event | Two or more prescriptions of vitamin K antagonists or thrombocyte aggregation inhibitors |
| De Vries et al [21] | Netherlands | Incident cardiovascular event | Two or more prescriptions of thrombocyte aggregation inhibitors |

**References**

1. Larsen J, Andersen M, Kragstrup J, Gram LF. High persistence of statin use in a Danish population: compliance study 1993-1998. Br J Clin Pharmacol. 2002;53:375-8.

2. Abraha I, Montedori A, Stracci F, Rossi M, Romagnoli C. Statin compliance in the Umbrian population. Eur J Clin Pharmacol. 2003;59:659-61.

3. Biagi C, Poluzzi E, Roberto G, Puccini A, Vaccheri A, D’Alessandro R, et al. Pattern of triptan use and cardiovascular coprescription: a pharmacoepidemiological study in Italy. Eur J Clin Pharmacol. 2011;67:1283-9.

4. Christiansen CF, Christensen S, Mehnert F, Cummings SR, Chapurlat RD, Sorensen HR. Glucocorticoid use and risk of atrial fibrillation or flutter: a population-based, case-control study. Arch Intern Med. 2009;169:1677-83.

5. Friis S, Poulsen AH, Johnsen SP, McLaughlin JK, Fryzek JP, Dalton SO, et al. Cancer risk among statin users: a population-based cohort study. Int J Cancer. 2005;114:643-7.

6. Latry P, Molimard M, Dedieu B, Couffinhal T, Begaud B, Martin-Latry K. Adherence with statins in a real-life setting is better when associated cardiovascular risk factors increase: a cohort study. BMC Cardiovasc Disord. 2011;11:46.

7. Bak S, Tsiropoulos I, Kjaersgaard JO, Andersen M, Mellerup E, Hallas J, et al. Selective serotonin reuptake inhibitors and the risk of stroke: a population-based case-control study. Stroke. 2002;33:1465-73.

8. Bak S, Andersen M, Tsiropoulos I, Garcia Rodriguez LA, Hallas J, Christensen K, et al. Risk of stroke associated with nonsteroidal anti-inflammatory drugs: a nested case-control study. Stroke. 2003;34:379-386.

9. Maitland-van der Zee AH, Klungel OH, Stricker BH, van der Kuip DA, Witteman JC, Hofman A, et al. Repeated nitrate prescriptions as a potential marker for angina pectoris. A comparison with medical information from the Rotterdam Study. Pharm World Sci. 2003;25:70-72.

10. Filippi A, Gensini G, Bignanimi AA, Sabatini A, Mazzaglia G, Cricelli C. Management of patients with suspected angina, but without known myocardial infarction: a cross-sectional survery. Br J Gen Pract. 2004;54:429-33.

11. Williams D, Bennett K, Feely J. Evidence for an age and gender bias in the secondary prevention of ischaemic heart disease in primary care. Br J Clin Pharmacol. 2003;55:604-8.

12. Johnson JA, Majumdar SR, Simpson SH, Toth EL. Decreased mortality associated with the use of metformin compared with sulfonylurea monotherapy in type 2 diabetes. Diabetes Care. 2002;25:2244-8.

13. Teeling M, Bennett K, Feely J. The influence of guidelines on the use of statins: analysis of prescribing trends 1998-2002. Br J Clin Pharmacol. 2005;59:227-32.

14. Wan Md Adnan WA, Zaharan NL, Bennet K, Wall CA. Trends in co-prescribing of angiotensin converting enzyme inhibitors and angiotensin receptor blockers in Ireland. Br J Clin Pharmacol. 2011;71:458-66.

15. Rasmussen JN, Gislason GH, Abildstrom SZ, Rasmussen S, Gustafsson I, Buch P, et al. Statin use after myocardial infarction: a nationwide study in Denmark. Br J Clin Pharmacol. 2005;60:150-158.

16. Meijvis SC, Cornips MC, Voorn GP, Souverein PC, Endeman H, Biesma DH, et al. Microbial evaluation of proton-pump inhibitors and the risk of pneumonia. Eur Respir J. 2011;38:1165-72.

17. Van de Steeg-van Gompel CH, Wensing M, Braspenning J, De Smet PA. The usefulness of antiplatelet prescriptions for the identification of patients with atherothrombosis in primary care: A Dutch cross-sectional study. J Eval Clin Pract. 2012;18:866-71.

18. Cossman RE, Cossman JS, James WL, Blanchard T, Thomas RK, Pol LG, et al. Evaluating heart disease prescriptions-filled as proxy for heart disease prevalence rates. J Health Human Serv Admin. 2008;30:503-28.

19. Gray J, Majeed A, Kerry S, Rowlands G. Identifying patients with ischaemic heart disease in general practice: Cross sectional study of paper and computerised medical records. BMJ. 2000;321:548-50.

20. Donnan PT, Dougall HT, Sullivan FM. Optimal strategies for identifying patients with myocardial infarction in general practice. Fam Pract. 2003;20:706-10.

21. De Vries FM, Denig P, Vegter S, Bos HJ, Postma MJ, Hak E. Does a cardiovascular event change adherence to statin treatment in patients with type 2 diabetes? A matched cohort design. Curr Med Res Opin. 2015;31:595-602.

**Appendix B. Table B1. Identifying major CVD hospitalizations^a^**

| **ICPC code / drug prescription** | **TP** | **FP** | **TN** | **FN** | **Sens** (95% CI) | **Spec**  (95% CI) | **PPV** (95% CI) | **NPV**  (95% CI) |
| --- | --- | --- | --- | --- | --- | --- | --- | --- |
| **IHD/cerebrovascular events** | | | | | | | | |
| GP codes | 251 | 402 | 10165 | 289 | 46  (42-51) | 96  (96-97) | 38  (35-42) | 97  (97-98) |
| GP codes / nitrates | 372 | 1038 | 9529 | 168 | 69  (65-73) | 90  (90-91) | 26  (24-29) | 98  (98-99) |
| GP codes / nitrates / platelet aggregation inhibitors | 495 | 3328 | 7239 | 45 | 92  (89-94) | 69  (68-69) | 13  (12-14) | 99  (99-100) |
| GP codes / nitrates / platelet aggregation inhibitors / vitamin k antagonists | 513 | 4070 | 6497 | 27 | 95  (93-97) | 61  (61-62) | 11  (10-12) | 100  (99-100) |
| **IHD events** | | | | | | | | |
| GP codes | 160 | 317 | 10417 | 213 | 43  (38-48) | 97  (97-97) | 34  (29-38) | 98  (98-98) |
| GP codes / nitrates | 268 | 989 | 9745 | 105 | 72  (67-76) | 91  (90-91) | 21  (19-24) | 99  (99-99) |
| GP codes / nitrates / platelet aggregation inhibitors | 345 | 3435 | 7299 | 28 | 92  (89-95) | 68  (67-69) | 9  (8-10) | 100  (99-100) |
| GP codes / nitrates / platelet aggregation inhibitors / vitamin k antagonists | 353 | 4204 | 6530 | 20 | 95  (92-97) | 61  (60-62) | 8  (7-9) | 100  (100-100) |
| **Cerebrovascular events** | | | | | | | | |
| GP codes | 85 | 118 | 10853 | 51 | 63  (54-71) | 99  (99-99) | 42  (35-49) | 100  (99-100) |
| GP codes / platelet aggregation inhibitors | 124 | 3382 | 7589 | 12 | 91  (85-95) | 69  (68-70) | 4  (3-4) | 100  (100-100) |
| GP codes / platelet aggregation inhibitors / vitamin k antagonists | 128 | 4309 | 6662 | 8 | 94  (89-97) | 61  (60-62) | 3  (2-3) | 100  (100-100) |

*Abbreviations:* IHD, ischaemic heart disease; CVD, cardiovascular disease; GP, general practitioner; TP, true positive; FP, false positive; TN, true negative; FN, false negative; Sens, sensitivity; Spec, specificity; PPV, positive predictive value; NPV, negative predictive value.

^a^ Patients from GPs with a possible relatively poor registration and those who have a GP in an area for which the closest hospital did not provide complete data to the DHD register during the entire study period are excluded for these analyses.

**Appendix C. Table C1. Identifying a history of major CVD using GP diagnoses as a reference standard**

| **Drug (at least 1 prescription)** | **TP** | **FP** | **TN** | **FN** | **Sens** (95% CI) | **Spec** (95% CI) | **PPV** (95% CI) | **NPV** (95% CI) |
| --- | --- | --- | --- | --- | --- | --- | --- | --- |
| **IHD/cerebrovascular history** | | | | | | | | |
| Diuretics (excl. thiazides) | 1301 | 5976 | 8049 | 1133 | 53  (51-55) | 57  (57-58) | 18  (17-19) | 88  (87-88) |
| Thiazides | 478 | 2776 | 11249 | 1956 | 20  (18-21) | 80  (80-81) | 15  (13-16) | 85  (85-86) |
| Beta blocking agents | 1527 | 4446 | 9579 | 907 | 63  (61-65) | 68  (68-69) | 26  (24-27) | 91  (91-92) |
| Calcium channel blockers | 821 | 2620 | 11405 | 1613 | 34  (32-36) | 81  (81-82) | 24  (22-25) | 88  (87-88) |
| Agents acting on the renin-angiotensin system | 1674 | 7772 | 6253 | 760 | 69  (67-71) | 45  (44-45) | 18  (17-19) | 89  (88-90) |
| Statins | 1933 | 9044 | 4981 | 501 | 79  (78-81) | 36  (35-36) | 18  (17-18) | 91  (90-92) |
| Fibrates | 66 | 258 | 13767 | 2368 | 3  (2-3) | 98  (98-98) | 20  (16-25) | 85  (85-86) |
| Nicotinic acid and derivatives | 15 | 26 | 13999 | 2419 | 1  (0-1) | 100  (100-100) | 37  (22-53) | 85  (85-86) |
| Other lipid modifying agents | 134 | 407 | 13618 | 2300 | 6  (5-6) | 97  (97-97) | 25  (21-29) | 86  (85-86) |
| Vitamin K antagonists | 424 | 1029 | 12996 | 2010 | 17  (16-19) | 93  (92-93) | 29  (27-32) | 87  (86-87) |
| Platelet aggregation inhibitors | 1696 | 2500 | 11525 | 738 | 70  (68-72) | 82  (82-83) | 40  (39-42) | 94  (94-94) |
| Nitrates | 487 | 441 | 13584 | 1947 | 20  (18-22) | 97  (97-97) | 52  (49-56) | 87  (87-88) |
| Vitamin K antagonists + Platelet aggregation inhibitors | 75 | 88 | 13937 | 2359 | 3  (2-4) | 99  (99-99) | 46  (38-54) | 86  (85-86) |
| Vitamin K antagonists / Platelet aggregation inhibitors | 2045 | 3441 | 10584 | 389 | 84  (83-85) | 75  (75-76) | 37  (36-39) | 96  (96-97) |
| (Vitamin K antagonists / Platelet aggregation inhibitors) + Nitrates | 455 | 363 | 13662 | 1979 | 19  (17-20) | 97  (97-98) | 56  (52-59) | 87  (87-88) |
| Vitamin K antagonists / Platelet aggregation inhibitors / Nitrates | 2077 | 3519 | 10506 | 357 | 85  (84-87) | 75  (75-76) | 37  (36-38) | 97  (96-97) |

*Abbreviations:* IHD, ischaemic heart disease; CVD, cardiovascular disease; GP, general practitioner; TP, true positive; FP, false positive; TN, true negative; FN, false negative; Sens, sensitivity; Spec, specificity; PPV, positive predictive value; NPV, negative predictive value.

**Appendix D. Table D1. Identifying a history of major CVD using GP diagnoses as a reference standard^a^**

| **Drug** | **TP** | **FP** | **TN** | **FN** | **Sens** (95% CI) | **Spec** (95% CI) | **PPV** (95% CI) | **NPV** (95% CI) |
| --- | --- | --- | --- | --- | --- | --- | --- | --- |
| **IHD/cerebrovascular history , 1 prescription** | | | | | | | | |
| Vitamin K antagonists | 424 | 1029 | 12996 | 2010 | 17  (16-19) | 93  (92-93) | 29  (27-32) | 87  (86-87) |
| Platelet aggregation inhibitors | 1696 | 2500 | 11525 | 738 | 70  (68-72) | 82  (82-83) | 40  (39-42) | 94  (94-94) |
| Nitrates | 487 | 441 | 13584 | 1947 | 20  (18-22) | 97  (97-97) | 52  (49-56) | 87  (87-88) |
| Vitamin K antagonists / Platelet aggregation inhibitors | 2045 | 3441 | 10584 | 389 | 84  (83-85) | 75  (75-76) | 37  (36-39) | 96  (96-97) |
| Vitamin K antagonists / Platelet aggregation inhibitors / Nitrates | 2077 | 3519 | 10506 | 357 | 85  (84-87) | 75  (75-76) | 37  (36-38) | 97  (96-97) |
| **IHD/cerebrovascular history, 2 prescriptions** | | | | | | | | |
| Vitamin K antagonists | 405 | 969 | 13056 | 2029 | 17  (15-18) | 93  (93-94) | 29  (27-32) | 87  (86-87) |
| Platelet aggregation inhibitors | 1650 | 2383 | 11642 | 784 | 68  (66-70) | 83  (82-84) | 41  (39-42) | 94  (93-94) |
| Nitrates | 386 | 386 | 13639 | 2048 | 16  (14-17) | 97  (97-98) | 50  (46-54) | 87  (86-87) |
| Vitamin K antagonists / Platelet aggregation inhibitors | 1991 | 3290 | 10735 | 443 | 82  (80-83) | 77  (76-77) | 38  (36-39) | 96  (96-96) |
| Vitamin K antagonists / Platelet aggregation inhibitors / Nitrates | 2025 | 3349 | 10676 | 409 | 83  (82-85) | 76  (75-77) | 38  (36-39) | 96  (96-96) |
| **IHD/cerebrovascular history, 3 prescriptions** | | | | | | | | |
| Vitamin K antagonists | 360 | 859 | 13166 | 2074 | 15  (13-16) | 94  (93-94) | 30  (27-32) | 86  (86-87) |
| Platelet aggregation inhibitors | 1577 | 2213 | 11812 | 857 | 65  (63-67) | 84  (84-85) | 42  (40-43) | 93  (93-94) |
| Nitrates | 351 | 300 | 13725 | 2083 | 14  (13-16) | 98  (98-98) | 54  (50-58) | 87  (86-87) |
| Vitamin K antagonists / Platelet aggregation inhibitors | 1895 | 3033 | 10992 | 539 | 78  (76-79) | 78  (78-79) | 38  (37-40) | 95  (95-96) |
| Vitamin K antagonists / Platelet aggregation inhibitors / Nitrates | 1940 | 3096 | 10929 | 494 | 80  (78-81) | 78  (77-79) | 39  (37-40) | 96  (95-96) |

*Abbreviations:* IHD, ischaemic heart disease; CVD, cardiovascular disease; GP, general practitioner; TP, true positive; FP, false positive; TN, true negative; FN, false negative; Sens, sensitivity; Spec, specificity; PPV, positive predictive value; NPV, negative predictive value.

^a^ Evaluating influence of number of prescriptions to be regarded as a positive test

**Appendix E. Table E1. Identifying a history of major CVD using GP diagnoses as a reference standard^a^**

| **Drug (at least 1 prescription)** | **TP** | **FP** | **TN** | **FN** | **Sens** (95% CI) | **Spec** (95% CI) | **PPV** (95% CI) | **NPV** (95% CI) |
| --- | --- | --- | --- | --- | --- | --- | --- | --- |
| **IHD/cerebrovascular history** | | | | | | | | |
| Vitamin K antagonists | 309 | 614 | 8140 | 1493 | 17  (15-19) | 93  (92-94) | 33  (30-37) | 85  (84-85) |
| Platelet aggregation inhibitors | 1245 | 1485 | 7269 | 557 | 69  (67-71) | 83  (82-84) | 46  (44-47) | 93  (92-93) |
| Nitrates | 347 | 242 | 8512 | 1455 | 19  (17-21) | 97  (97-98) | 59  (55-63) | 85  (85-86) |
| Vitamin K antagonists / Platelet aggregation inhibitors | 1498 | 2058 | 6696 | 304 | 83  (81-85) | 76  (76-77) | 42  (40-44) | 96  (95-96) |
| Vitamin K antagonists / Platelet aggregation inhibitors / Nitrates | 1519 | 2104 | 6650 | 283 | 84  (83-86) | 76  (75-77) | 42  (40-44) | 96  (95-96) |

*Abbreviations:* IHD, ischaemic heart disease; CVD, cardiovascular disease; GP, general practitioner; TP, true positive; FP, false positive; TN, true negative; FN, false negative; Sens, sensitivity; Spec, specificity; PPV, positive predictive value; NPV, negative predictive value.

^a^ Patients from GPs with a possible relatively poor registration and those who have a GP in an area for which the closest hospital did not provide complete data to the DHD register during the entire study period are excluded for these analyses.

**Appendix F. Table F1. Identifying incident major CVD within -30 to 90 days of event^a,b^**

| **Drug prescription** | **TP** | **FP** | **TN** | **FN** | **Sens** (95% CI) | **Spec** (95% CI) | **PPV** (95% CI) | **NPV**  (95% CI) |
| --- | --- | --- | --- | --- | --- | --- | --- | --- |
| **GP/Hosp IHD/cerebrovascular events^c^** | | | | | | | | |
| Vitamin K antagonists | 10 | 200 | 9457 | 392 | 2  (1-5) | 98  (98-98) | 5  (2-9) | 96  (96-96) |
| Platelet aggregation inhibitors | 101 | 330 | 7425 | 120 | 46  (39-53) | 96  (95-96) | 23  (20-28) | 98  (98-99) |
| Nitrates | 47 | 192 | 9784 | 319 | 13  (10-17) | 98  (98-98) | 20  (15-25) | 97  (96-97) |
| Vitamin K antagonists / Platelet aggregation inhibitors | 90 | 409 | 6512 | 76 | 54  (46-62) | 94  (94-95) | 18  (15-22) | 99  (99-99) |
| Vitamin K antagonists / Platelet aggregation inhibitors / Nitrates | 92 | 425 | 6424 | 69 | 57  (49-65) | 94  (93-94) | 18  (15-21) | 99  (99-99) |
| **Hosp IHD/cerebrovascular events^d^** | | | | | | | | |
| Vitamin K antagonists | 9 | 203 | 9639 | 208 | 4  (2-8) | 98  (98-98) | 4  (2-8) | 98  (98-98) |
| Platelet aggregation inhibitors | 74 | 363 | 7493 | 46 | 62  (52-70) | 95  (95-96) | 17  (14-21) | 99  (99-100) |
| Nitrates | 36 | 207 | 9948 | 151 | 19  (14-26) | 98  (98-98) | 15  (11-20) | 99  (98-99) |
| Vitamin K antagonists / Platelet aggregation inhibitors | 65 | 440 | 6553 | 29 | 69  (59-78) | 94  (93-94) | 13  (10-16) | 100  (99-100) |
| Vitamin K antagonists / Platelet aggregation inhibitors / Nitrates | 68 | 456 | 6462 | 24 | 74  (64-83) | 93  (93-94) | 13  (10-16) | 100  (99-100) |

*Abbreviations:* IHD, ischaemic heart disease; CVD, cardiovascular disease; GP, general practitioner; Hosp, hospitalization; TP, true positive; FP, false positive; TN, true negative; FN, false negative; Sens, sensitivity; Spec, specificity; PPV, positive predictive value; NPV, negative predictive value.

^a^ Patients from GPs with a possible relatively poor registration and those who have a GP in an area for which the closest hospital did not provide complete data to the DHD register during the entire study period are excluded for these analyses.

^b^ Prescriptions are considered to be in agreement with actual events if being prescribed between 30 days before to 90 days after the date of the major IHD or cerebrovascular event.

^c^ Using hospitalizations or general practitioners diagnoses as reference standard.

^d^ Using only hospitalizations as reference standard.
